# A case report of cryoablation and electrochemotherapy in kidney cancer

**DOI:** 10.1097/MD.0000000000027730

**Published:** 2021-11-12

**Authors:** Giovanni Mastrandrea, Carmelo Laface, Vito Fazio, Marco Lopetuso, Gianmarco Falagario, Pasquale Molinari, Girolamo Ranieri, Cosmo Damiano Gadaleta

**Affiliations:** a Anaesthesia, Resuscitation and Postoperative Intensive Care Unit, IRCCS Istituto Tumori “G. Paolo II”, Bari, Italy; b Interventional and Medical Oncology Unit, IRCCS Istituto Tumori “G. Paolo II”, Bari, Italy; c Department of Biomedical Sciences and Clinical Oncology, University of Bari Aldo Moro, Bari, Italy; d Department of Basic Medical Sciences, Neuroscience and Sense Organs, University of Bari Aldo Moro, Bari, Italy.

**Keywords:** cryoablation, electrochemotherapy, kidney cancer

## Abstract

**Rationale:**

According to scientific literature, cryoablation (CA) and electrochemotherapy (ECT) have been used for the treatment of small renal masses. However, no data have been published regarding the combination of these techniques as therapy of primary kidney cancers. Therefore, we report the case of an old woman affected by localized kidney cancer and discuss the potential therapeutic application of CA combined with subsequent deep ECT in this setting.

**Patient concerns:**

An 85 years-old-woman was evaluated because of a localized kidney cancer. Her background history included long-time hypertension and diabetes mellitus in drug treatment.

**Diagnoses:**

In February 2018, the follow-up contrast enhancement computed tomography (ceCT) documented a suspected 18×10 mm metastasis at the lower right lobe of the lung. The ceCT also showed a suspected primary malignancy of 25×18 mm at right kidney.

**Interventions:**

The kidney cancer was treated with a two-phase procedure: percutaneous CA and subsequent deep ECT.

**Outcomes:**

Patient obtained a complete response according to modified Response Evaluation Criteria in Solid Tumors, without renal function or quality of life impairment. No procedure-related complications were observed. Moreover, a shorter period of hospitalization and convalescence were needed respect to standard surgery. No sign of relapse was observed during follow-up period.

**Lessons:**

This combined strategy proved to be safe and effective. Moreover, the application of these blended loco-regional techniques showed several other advantages such as reduced hospitalization and a shorter period of convalescence respect to standard surgery.

## 1 Introduction

Kidney cancer is the 10^th^ most common cancer in Italy. The incidence correlates with age, in fact, the highest incidence is during the 8^th^ decade of life. The 5-year survival is almost 70% with a strong age gradient: 5-year survival goes from 87% in the 15–44 years range to 56% in older people (> 75 years). At diagnosis, kidney cancer is confined to the kidney in 55% of cases, locally advanced in 19% of cases or with synchronous metastases in 25–30% of cases.^[1]^

Surgery is the standard treatment for localized kidney cancer. Surgical options are radical nephrectomy and nephron-sparing surgery (NSS).^[2]^ Active surveillance or ablative procedures (cryoablation (CA) and radiofrequency ablation (RFA) are reserved for selected cases; the limited data available show distant disease-free survival comparable to surgery, however suggesting an increased risk of locoregional progression. Kunkle et al^[3]^ in a meta-analysis compared NSS, CA, RFA and observation for small renal masses. They analysed 99 studies for a total of 6,471 lesions (for each treatment modality mean tumor size was 3.40, 2.56, 2.69 and 3.04 cm for NSS, CA, RFA and active surveillance, respectively). CA and RFA documented significantly increased local progression rates compared to NSS. However, no statistical differences were detected in the incidence of metastatic progression regardless of whether lesions were excised, ablated or observed. Whitson et al^[4]^ in a retrospective cohort study evaluated a total of 8818 small renal lesions ≤ 4 cm treated with NSS or ablation. They showed that patients underwent ablation had a twofold increase in the risk of kidney cancer death; however, at 5 years the absolute difference is small, and may only be realized by patients with long life expectancies.

With regards to other loco-regional techniques such as electrochemotherapy (ECT), in literature, there are only a few case reports regarding the treatment of small renal masses.^[5]^

To the best of our knowledge, there are few data published on the combination of loco-regional approaches for treatment of primary kidney cancer. Therefore, we report the case of an old woman affected by localized kidney cancer underwent to percutaneous CA and then deep percutaneous ECT in alternative to surgery, evaluating safety and efficacy of this combination strategy.

Both of these locoregional therapies have the advantage of being mini-invasive, repeatable and not exclusive. In our study, we successfully use them in sequence for the treatment in two phases of the same renal lesion.

## 2 Case report

An 85 years-old-woman was evaluated because of a localized kidney cancer. Her background history included long-time hypertension and diabetes mellitus in drug treatment. In 2015, she also had a rectal cancer that has been under neoadjuvant chemotherapy with Capecitabine combined to radiotherapy, proctectomy and subsequent adjuvant treatment with Capecitabine. In the following years, the patient performed six-monthly follow-up by thorax-abdomen contrast enhancement computed tomography (ceCT). In February 2018, the follow-up ceCT documented a suspected 18×10 mm metastasis at the lower right lobe of the lung. The ceCT also showed a suspected primary malignancy of 25×18 mm at right kidney (cT1a according to the American Joint Committee on Cancer 8^th^ edition (American Joint Committee-Tumour, Node, Metastasis) classification of kidney cancer).

**Figure 1:**
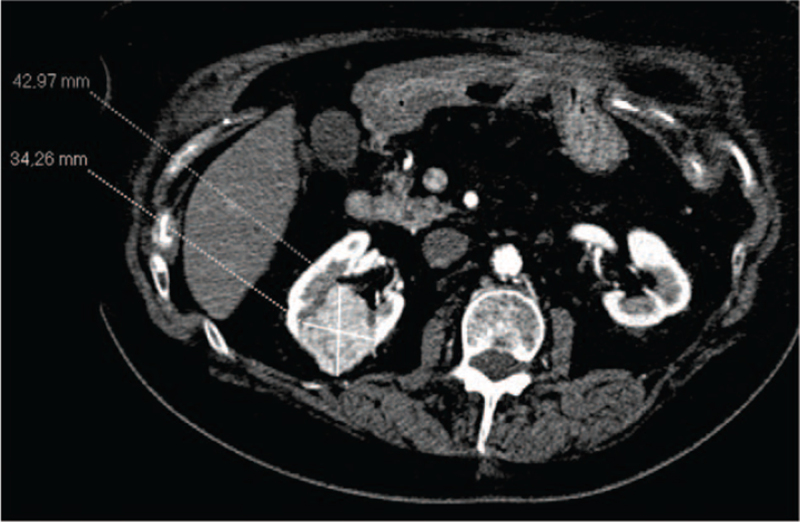
Abdomen CT scan showing the measurement of the hypervascular tumor lesion in the right kidney.

**Figure 2:**
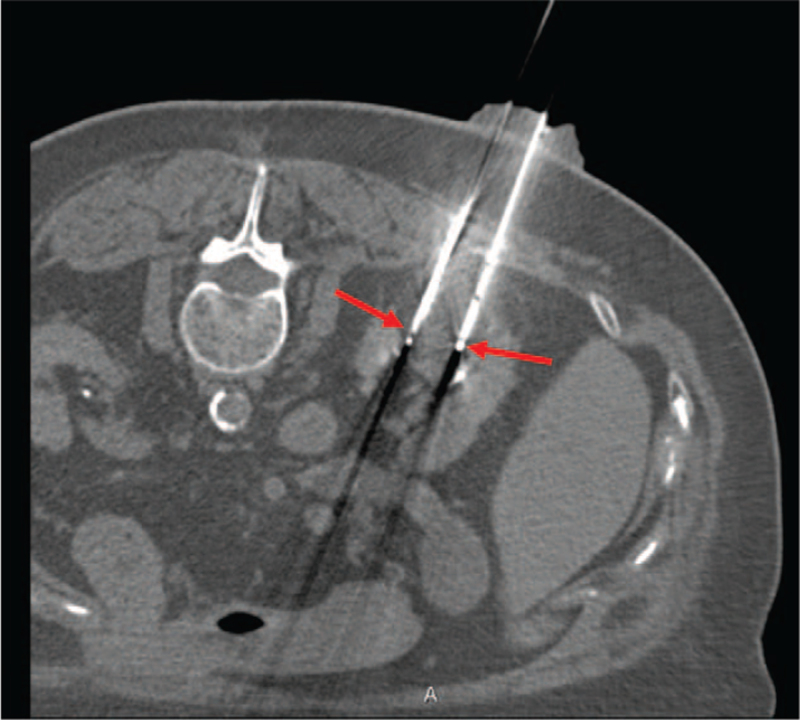
Intraoperative abdomen CT scan: the red arrows indicate two cryoprobes inserted into the target lesion.

**Figure 3:**
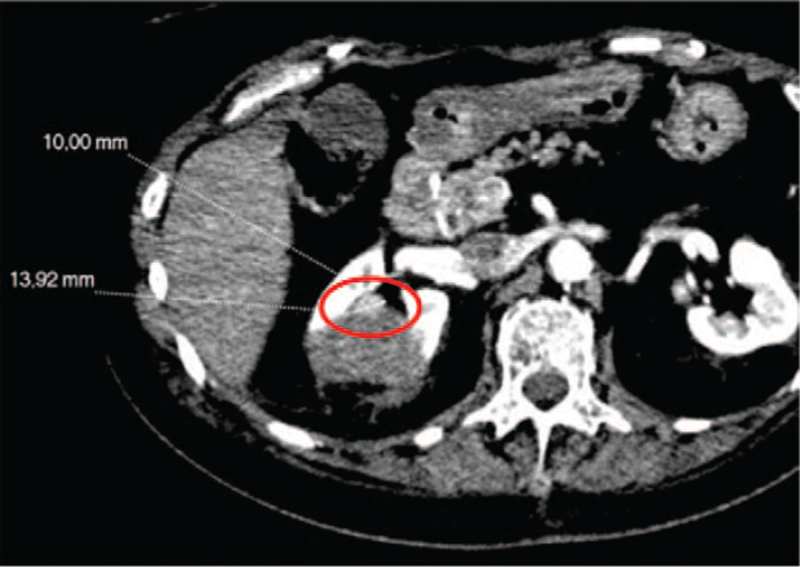
Abdomen CT scan post cryoablation: the red circle identifies the residual disease. Please, note the intratumoral arterial enhancement near to renal pelvis.

**Figure 4:**
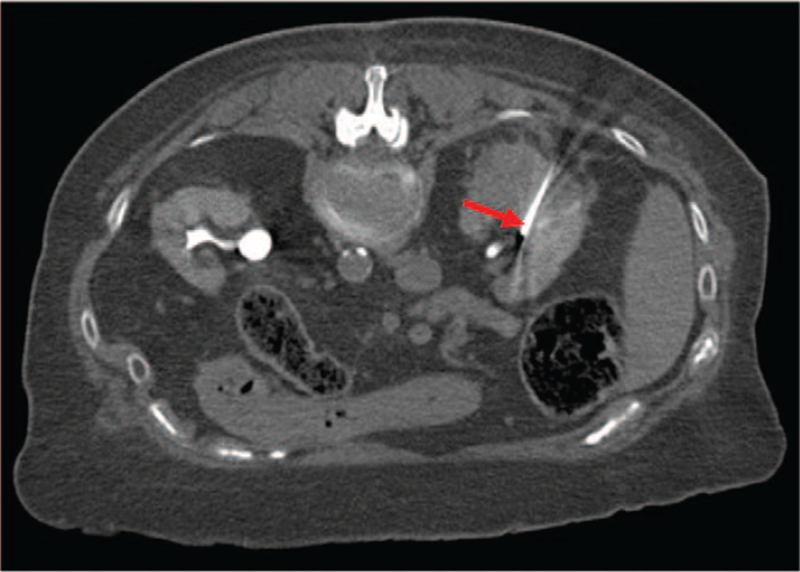
Abdomen CT scan during ECT treatment: the red arrow indicates one of the needle electrodes inserted into the residual lesion.

**Figure 5:**
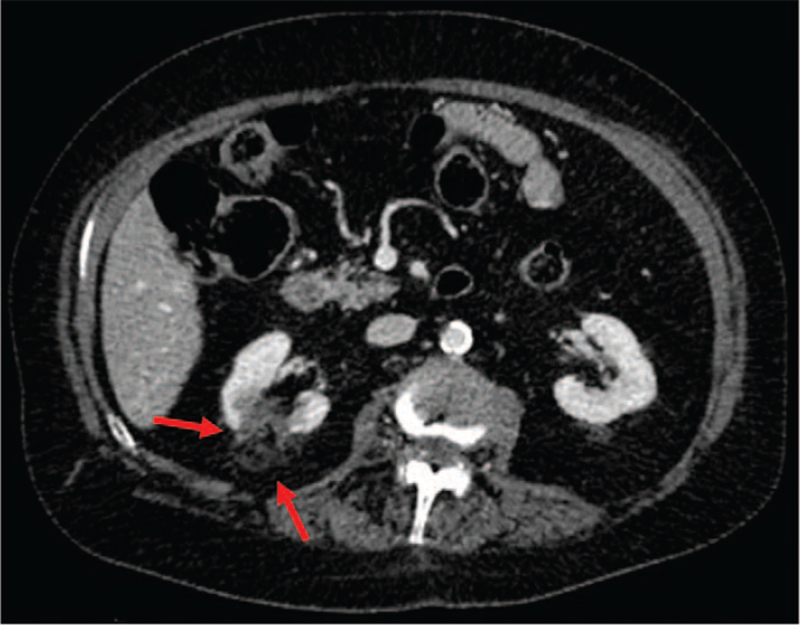
Post-treatments CT demonstrating complete response according to mRECIST. Please, note the disappearance of intratumoral vascular enhancement in the target lesion.

With regards to Positron Emission Tomography, the Standardized Uptake Value was 2.8 for lung lesion while no uptake of 18F-Fluorodeoxyglucose was observed in the renal lesion. It is well established the most part of localized kidney cancer doesn’t show 18F-Fluorodeoxyglucose uptake. In accordance with the rectal cancer history, the different metabolic activity of the two tumor lesions and the need to establish the therapeutic program, the patient was subjected to biopsy of both lesions. Lung biopsy was diagnostic for rectal adenocarcinoma metastasis, while renal biopsy confirmed a primitive carcinoma with unclear differentiation between papillary and chromophobic form and G2 pattern. At diagnosis, the patient had a grade 2 Performance Status according to Eastern Cooperative Oncology Group, III stage of chronic kidney disease based on Kidney Disease Outcomes Quality Initiative and a medium-high anesthesiological risk based on American Society of Anesthesiologists Physical Status Classification System (American Society of Anesthesiologists III due to poorly controlled diabetes mellitus and hypertension). Right nephrectomy and lung metastasectomy were proposed informing about high surgical risk of death, intra- and post-operative complications. The patient refused any surgical program. She headed to our attention in September 2018 when the ceCT showed an increased size of the lung metastasis (25 × 16 mm versus 18×10 mm) and of the kidney cancer (43×34 mm versus 25×18 mm; cT1b) [Fig. 1]. Therefore, based on the high surgical risk and the patient's wishes, we suggested loco-regional treatments in alternative to surgery. In December 2018, after obtained informed consent, she was subjected to microwave ablation of the lung metastasis. The procedure was carried out employing two successive 5 min infixions, respectively with 30 and 60 Watt emissions. In February 2019, the kidney cancer was treated with a two-phase procedure: percutaneous CA and subsequent deep ECT. The patient underwent balanced general anaesthesia during the procedures. Muscle relaxation made the interventional radiologist's approach easier. Percutaneous CA was performed through CT-guided implantation of 4 Ice Road cryoprobes (BTG, Boston Scientific) in the right kidney lesion and 2 subsequent CA cycles (standard 10 min freeze, 8 min thaw, 10 min freeze treatment for each cycle) were performed [Fig. 2]. Subsequently, one cryoprobe was inserted in the more caudal side of the lesion and other 2 subsequent CA cycles (the first of 8 min and the second of 10 min) were executed. To ensure that the tumour was covered with ice, CT scans were performed after 4 and 8 min during the treatment session. The whole procedure took 1 hour and 30 min, without any complications. An abdominal ceCT was performed after 30 days from the procedure showing a complete devascularization of the cryoablated area, except for a small conical-shaped renal tissue of 14×10 mm [Fig. 3]. The next day, after obtained informed consent, the patient underwent ECT, using an ECT system with electric pulses generated by Cliniporator Vitae (IGEA S.p.A). The lesion was only visible on post-contrast CT imaging. A total of four electrodes of 17G were placed into the target area, under CT guidance, for covering all the area and to spare as much normal parenchyma as possible [Fig. 4]. An intravenous administration of bleomycin (15.000 UI/m^2^) was performed 8 min before the electrodes were activated with R-wave electrocardiogram synchronization via AccuSync 42, an external R-wave-triggering device (AccuSync, USA). During ECT, no complications were reported. The patient was then discharged 2 days after the procedure. In June 2019, the ceCT showed complete tumor necrosis without residual viable tumor tissue in the treated area [Fig. 5]. Therefore, we obtained a complete response according to modified Response Evaluation Criteria in Solid Tumors, the disappearance of any intratumoral arterial enhancement in the target lesion.^[9,10]^ Thus, the patient started follow-up through ceCT every 6 months and, up to date, no sign of relapse was observed. Besides, the values of renal function have always remained within the limits.

## 3 Discussion

Percutaneous CA is a minimally invasive technique able to destroy targeted tissue through the application of extreme cold.^[6]^ Rapid expansion of pressured argon gas within the cryoprobe leads to very low temperature (-40°C) of the tissue and the formation of an expanding iceball on the cryoprobe tip. Immediately adjacent to the cryoprobe, rapid freezing leads to intracellular ice crystal formation causing mechanical trauma to cell membranes and subsequent apoptosis. The real-time control allows to monitor the formation of the iceball, to avoid unintentional harm to the critical structures and to ensure complete tumor coverage. Nowadays renal CA is considered a treatment option for selected patients affected by small renal masses (≤4 cm).^[3,4,11,12]^ This technique allows to preserve renal function with a high disease control. At the same time, it is characterized by a low complication rate, short hospitalization, and higher compliance by patients. Early CT imaging is helpful to evaluate the treatment efficacy. Post-operative follow-up is structured at specific intervals and can be managed according to the degree of suspicion of inadequate treatment. To be specific, incomplete treatment is defined as an enhancing lesion at the site of ablation within the first 3 months while late local recurrence is defined as an enhancing lesion at the site of ablation following at least one clear imaging study.^[11]^ Our clinical case corresponds to an example of incomplete treatment. However, we excluded another CA as well as other ablation techniques due to the anatomical site of the residual disease, for sparing normal parenchyma. To be specific, the tumor lesion was placed near to renal pelvis so another ablation treatment would have invalidated renal function in a patient already suffering from moderate chronic kidney disease.

ECT is a non-thermal technique based on the electroporation of tissue cells and the concomitant administration of chemotherapeutic drugs.^[7]^ The application of an external electrical field to a cell membrane induces transient permeability and favours the cell exposure to chemotherapeutic drugs, which are highly cytotoxic but poorly permeating such as bleomycin.^[8]^ ECT is usually employed for the treatment of cutaneous metastases from melanoma/non-melanoma skin cancer or breast cancer but also bone, liver metastases, or soft tissue sarcoma.^[8]^ ECT focuses the cytotoxic effects of the chemotherapeutic drugs on target tissues which are exposed to the electrical pulses. This local potentiation of chemotherapy allows to reduce the systemic side effects and to spare adjacent normal tissue. ECT has been already used to treat local recurrence of kidney cancer when other ablative techniques are not suitable.^[5]^ On these bases, we proposed to treat the residual disease through deep percutaneous ECT with favourable outcome.

In detail, patient obtained high disease control without renal function or quality of life impairment. Moreover, a shorter period of hospitalization and convalescence were needed respect to standard surgery.

## 4 Conclusion

The presented case report suggests as the strategy to combine CA and subsequent ECT might be safe and effective for the treatment of primary kidney cancer in selected patients, in alternative to surgery.

The choice to employ CA as first procedure is due to the possibility to evaluate efficacy treatment after a short period from the procedure, unlike ECT. This allowed to reduce the time between the first and the second treatment minimizing the risk of tumor relapse.

Moreover, our data show as ECT might be useful to treat residual disease after CA sparing normal parenchyma. Further studies are necessary to better define the potentiality of this combination strategy and which patients could benefit in major.

## Author contributions

**Conceptualization:** Giovanni Mastrandrea, Girolamo Ranieri.

**Data curation:** Carmelo Laface.

**Investigation:** Giovanni Mastrandrea, Vito Fazio, Marco Lopetuso, Gianmarco Falagario, Pasquale Molinari.

**Methodology:** Girolamo Ranieri.

**Supervision:** Girolamo Ranieri, Cosmo Damiano Gadaleta.

**Validation:** Girolamo Ranieri, Cosmo Damiano Gadaleta.

**Writing – original draft:** Giovanni Mastrandrea, Marco Lopetuso.

**Writing – review & editing:** Carmelo Laface.

## References

[1] Chow WH Dong LM Devesa SS. Epidemiology and risk factors for kidney cancer. Nat Rev Urol 2010;7:245–57. 20448658 10.1038/nrurol.2010.46 PMC3012455

[2] Auffenberg GB Curry M Gennarelli R Blum KA Elkin E Russo P. Comparison of cancer specific outcomes following minimally invasive and open surgical resection of early stage kidney cancer from a National Cancer Registry. J Urol 2020;203:1094–100. 31913076 10.1097/JU.0000000000000741 PMC8498972

[3] Kunkle DA Egleston BL Uzzo RG. Excise, ablate or observe: the small renal mass dilemma — a meta-analysis and review. J Urol 2008;179:1227–34. 18280512 10.1016/j.juro.2007.11.047

[4] Whitson JM Harris CR Meng MV. Population-based comparative effectiveness of nephron-sparing surgery vs ablation for small renal masses. BJU Int 2012;110:1438–43. 22639860 10.1111/j.1464-410X.2012.11113.x

[5] Andresciani F Faiella E Altomare C Pacella G Beomonte Zobel B Grasso RF. Reversible electrochemotherapy (ECT) as a treatment option for local RCC recurrence in solitary kidney 2020;43:1091–4. 10.1007/s00270-020-02498-2 32415331

[6] Orsi F Varano G. Minimal invasive treatments for liver malignancies. Ultrason Sonochem 2015;27:659–67. 26050603 10.1016/j.ultsonch.2015.05.030

[7] Tafuto S von Arx C De Divitiis C. Electrochemotherapy as a new approach on pancreatic cancer and on liver metastases. Int J Surg 2015;21: (Suppl 1): S78–82. 26123385 10.1016/j.ijsu.2015.04.095

[8] Ranieri G Laface C Fazio V. Local treatment with deep percutaneous electrochemotherapy of different tumor lesions: pain relief and objective response results from an observational study. Eur Rev Med Pharmacol Sci 2020;24:7764–75. 32744703 10.26355/eurrev_202007_22279

[9] Lencioni R Llovet JM. Modified RECIST (mRECIST) assessment for hepatocellular carcinoma. Semin Liver Dis 2010;30:52–60. 20175033 10.1055/s-0030-1247132 PMC12268942

[10] Llovet JM Lencioni R. mRECIST for HCC: performance and novel refinements. J Hepatol 2020;72:288–306. 31954493 10.1016/j.jhep.2019.09.026 PMC12452114

[11] Ismail M Nielsen TK Lagerveld B. Renal cryoablation: multidisciplinary, collaborative and perspective approach. Cryobiology 2018;83:90–4. 29890126 10.1016/j.cryobiol.2018.06.002

[12] Thompson RH Atwell T Schmit G. Comparison of partial nephrectomy and percutaneous ablation for cT1 renal masses. Eur Urol 2015;67:252–9. doi: 10.1016/j.eururo.2014.07.021. 25108580 10.1016/j.eururo.2014.07.021

